# Editorial: T Cell Exhaustion

**DOI:** 10.3389/fimmu.2020.00920

**Published:** 2020-06-16

**Authors:** Cristina Bonorino, Giuliana Mognol

**Affiliations:** ^1^Universidade Federal de Ciencias da Saude de Porto Alegre, Departamento de Ciencias Basicas da Saude, Porto Alegre, Brazil; ^2^Department of Surgery, University of California, San Diego, San Diego, CA, United States; ^3^Bluestar Genomics, San Diego, CA, United States

**Keywords:** exhaustion, T cells, cancer, viral infection, PD-1 - PDL-1 axis

The term “exhaustion” has become one of the most ubiquitously employed terms in immunology, appearing in articles that range from infection to cancer and from innate immunity to immunological memory. Inspired by finishing an article distinguishing the features of exhaustion from those triggered by acute activation in a tumor model (1) and attending the 2018 AACR meeting, we decided to put together a Frontiers in Immunology Research Topic focusing on T cell exhaustion. The result is a collection of seven expert articles from authors that have contributed their work, and they discuss some most intriguing aspects of this phenomenon.

T cell exhaustion describes a state in which cells progressively decrease and finally cease to proliferate and function due to excessive antigenic stimulation in the absence of co-stimulation, and this is often found in chronic infection and cancer (2). As Pawelec et al. reminds us in his paper, exhaustion is often thought of as something negative—but this is not necessarily the case. Many times, exhaustion is a transient state that can be reversed by the activation of certain signaling pathways. It can thus constitute an effective physiological mechanism to maintain T cells in the repertoire, preventing continued division so T cells do not reach the Hayflick limit and undergo senescence.

Exhausted cells express high levels of inhibitory receptors, including CTLA-4, LAG-3, PD-1, and TIM-3. Checkpoint blockade inhibitors (blocking antibodies against these inhibitory receptors) show remarkable efficacy in reversing exhaustion and promoting tumor regression (3). This has resulted in a surge of intense investigation on the mechanisms that govern exhaustion.

In order to optimize strategies to reverse exhaustion, Guram et al. propose a threshold model of activation that comprises the necessary signaling and transcriptional requirements for T cell reactivation in anti-tumor responses. These requirements would not be restricted to T cells but would also be extended to APCs, as they are the ones providing the key regulatory signals to T cells.

APC activation can also be crucial to the generation of abscopal effects—described mainly in radiation therapy, when local destruction of the primary tumor is followed by regression of a distant tumor. Suek et al. raise the interesting point that abscopal effects can occur in therapies that activate the APC but do not destroy tumor cells and thus do not result in massive antigen release. They review data on the abscopal effects generated by intratumoral (rather than systemic or cutaneous) use of TLR9 agonists, which has yielded positive results in recent clinical studies, especially when associated with checkpoint blockade immunotherapy.

The use of checkpoint inhibitors in cancer therapy, although with unprecedented results, only elicits responses in a percentage of patients, indicating there is still much to understand about the immune synapse and the cells that engage in it. Which T cells are being targeted, and which ones can recover? Tumor-infiltrating T cells are highly heterogeneous, presenting different subpopulations, states of activation, and TCR usage. Cui et al. asked if the T cell repertoire could serve as a predictive marker of the immune response in cervical cancer. They found that TCR repertoire diversity was decreased in tumors vs. draining lymph nodes and that TCR usage of blood T cells in cancer patients was also less diverse that in cancer-free individuals. They propose a model in which exhaustion associates with low T cell TCR diversity. Menard et al. analyzed a massive expansion of double positive (DP) (CD4^+^CD8^+^) T cells inside renal cell carcinomas (RCC) that expressed exhaustion markers. These cells also presented a high degree of clonality, as seen by TCR repertoire sequencing. These DP cells may be specific for tumor antigens, as suggested by the expression of markers associated with antigen experience and memory phenotype, and thus could constitute major targets for reactivation by checkpoint blockade inhibitors.

Viral infections have still much to teach about T cell exhaustion. Saeidi et al. reviewed the importance of transcriptional as well as metabolic alterations for the optimal reactivation of exhausted T cells. Profound changes in the epigenetic profile and energy production are observed in response to PD-1/PD-L1 blockade. While activated T cells in viral infections use OXPHOS and upregulate mitochondrial mass, the exhausted, PD-1^+^ T cells switch to a glycolytic metabolism with low mitochondrial mass, and this can be improved by antioxidant and cytokine treatment. Still focusing on viral infections, the study by David et al. brings interesting data on the relative contribution of PD-1 vs. PD-L1 on CD8^+^T cells. Although most studies focus on the role of PD-1 and PD-L1 in the chronic phase of viral infection, few of them investigate the role of this pathway in the early phases of the anti-viral immune response. Moreover, it is commonly thought that the blockade of PD-1/PD-L1 targets PD-1 on the T cells and PD-L1 on the APC. In their model, PD-1 controlled response magnitude during acute infection, while PD-L1 on T cells performed quality control, meaning determining the type of T-cell function after co-stimulation. Deletion of either one of the molecules, however, affected proliferation, maturation, and apoptosis of effector cytotoxic T cells. Thus, knocking out PD-L1 on T cells, had a greater impact on T cell function than knocking out PD-1, leading to the increase in polyfunctional cells.

There is still a lot to be understood about the immune synapse, its impact on exhaustion, and the mechanisms affected by checkpoint blockade immunity to accurately predict and even increase response to immunotherapy. The authors that contributed to this special topic will certainly contribute to solving these questions in the future. We hope you enjoy the reading.

## Author Contributions

All authors listed have made a substantial, direct and intellectual contribution to the work, and approved it for publication.

## Conflict of Interest

The authors declare that the research was conducted in the absence of any commercial or financial relationships that could be construed as a potential conflict of interest.
